# Hepatocellular Carcinoma After HCV Eradication with Direct-Acting Antivirals: A Reappraisal Based on New Parameters to Assess the Persistence of Risk

**DOI:** 10.3390/cancers17061018

**Published:** 2025-03-18

**Authors:** Eduardo Fassio, Luis Colombato, Gisela Gualano, Soledad Perez, Miguel Puga-Tejada, Graciela Landeira

**Affiliations:** 1 Liver Section, Gastroenterology Service, Hospital Nacional Profesor Alejandro Posadas, El Palomar, Buenos Aires 1684, Argentina; draperezsoledad@gmail.com (S.P.); glandeira@intramed.net (G.L.); 2 Hospital Británico de Buenos Aires, Buenos Aires 1280, Argentina; colombato@gmail.com; 3 Hospital Regional Dr. Ramón Carrillo, Santiago del Estero 4200, Argentina; giselagualano@gmail.com; 4 Instituto Ecuatoriano de Enfermedades Digestivas, Guayaquil 090505, Ecuador; mpuga@ieced.ec

**Keywords:** hepatocellular carcinoma, hepatitis C, liver cirrhosis, surveillance, direct-acting antivirals, incidence of hepatocellular carcinoma

## Abstract

Approximately 95% of patients with chronic hepatitis C achieve viral eradication through direct-acting antiviral (DAA) treatment. Ensuing clinical benefits include halting liver fibrosis, thereby reducing the need for liver transplantation, and decreasing both liver-related and overall mortality. It is well established that, although ameliorated, the risk of developing hepatocellular carcinoma (HCC) persists, particularly among patients with pre-treatment advanced fibrosis/cirrhosis. Current guidelines recommend indefinite HCC surveillance in these patients. However, a recent Markov model evaluation shows that HCC surveillance is cost-effective only for patients with cirrhosis but not so for those with F3 fibrosis, a finding which points out the need to better define the risk of HCC in hepatitis C patients after cure and further characterize pre- and post-treatment factors that might affect the incidence of HCC in this setting. We reviewed the literature analyzing this aspect. Here we summarize the main findings: male gender and older age are independent predictors of increased risk of post-cure HCC development. Moreover, non-invasive tests for hepatic fibrosis, namely FIB4, APRI, and liver stiffness, measured before and after treatment and their post-therapy change, contribute to better stratifying the risk of HCC occurrence. Furthermore, low serum albumin, as well as an AFP above 7 ng/mL prior to and after DAA therapy, also constitute independent predictors of HCC development. Considering these findings, we propose to classify patients with HCV viral eradication and advanced fibrosis/cirrhosis into groups of low, medium, or high risk of HCC and to adopt adequate surveillance strategies for each group, including protocols for abbreviated magnetic resonance imaging (MRI) for those at the highest risk.

## Simple Summary

Direct-acting antivirals, DAAs, represent the most effective treatment for HCV infection, with clear-cut advantages over the interferon-based regimens, since they are orally administrated, applicable to the whole spectrum of HCV infection, including acute, chronic, hepatic, and extrahepatic, and effective in all liver fibrosis stages, reaching a viral eradication rate of 95% in a very short treatment period, with few and mild adverse events. After the eradication of HCV by means of DAAs, the risk of developing hepatocellular carcinoma significantly decreases, but it is not completely abolished among patients with advanced fibrosis (F3) or cirrhosis. In these latter patients, surveillance for HCC should continue. However, the occurrence of HCC is variable, and some authors have published that surveillance is not cost-effective among patients with initial mild liver fibrosis, i.e., stages F1 and F2, and even among those with F3 fibrosis, while in patients with cirrhosis (F4), HCC surveillance is mandatory. In this review, we analyze additional baseline features, as well as post-DAA variables, with an impact on the remaining risk of HCC occurrence. The current literature identifies patients at a low, medium, or high risk of HCC. In conclusion, we hereby advocate for a reappraisal of HCC screening methodology after DAA cure, adopting a different surveillance strategy based on the most precise evaluation of the post-cure remaining risk level for developing HCC.

## 1. Introduction

It is estimated that approximately 50 million persons are infected by HCV worldwide, causing a silent epidemic and a very important burden for health systems. For decades, chronic HCV infection has been the main etiology of chronic hepatitis in the West, the leading indication for liver transplantation in the United States of America (USA) [1] and other western countries, and the main cause of hepatocellular carcinoma (HCC) in Japan, Mediterranean Europe [2], the USA [1,3], and Latin America [4,5]. In the USA, since 2007, deaths from hepatitis C have outnumbered those related to HIV. Importantly, this occurs among middle-aged persons [5].

Most individuals who become infected with HCV do not resolve the infection spontaneously and will become chronic carriers. However, the natural history of chronic hepatitis C is highly variable and slowly progressive, with approximately 20–30% of patients progressing to cirrhosis within 20–30 years. There are factors that may increase the risk of progression to cirrhosis, such as older age at the time of infection, male sex, excessive alcohol intake, infection with genotype 3 HCV, coinfections with HIV or HBV, and the presence of diabetes or hepatic steatosis. The prognosis of a patient infected with HCV is associated with the severity of the liver damage, and the risk of presenting clinical outcomes, such as complications of cirrhosis or HCC, during follow-up is possible when the biopsy shows F3 fibrosis (bridging fibrosis) and very likely when there is F4 fibrosis (cirrhosis). On the contrary, patients with no fibrosis (F0) or with mild fibrosis (F1-F2) are not at risk and do not require further controls after HCV eradication.

Direct-acting antiviral (DAA) drugs have produced a true revolution in the treatment of chronic hepatitis C. Three main classes of DAAs are available, including polymerase inhibitors, protease inhibitors, and NS5A inhibitors. Combining two or three drugs that act in different steps in HCV viral replication significantly increases antiviral efficacy and reduces the incidence of substitutions associated with resistance. These new regimens achieved sustained virological response (SVR) in approximately 95% of patients with chronic hepatitis C, not only in registration trials [6,7,8,9] but also in real-life scenarios [10,11,12]. Because of its safety profile, the applicability of antiviral therapy has been expanded to the stage of decompensated cirrhosis [13,14]. Two pangenotypic regimens are the most used as first-line therapy, sofosbuvir/velpatasvir and glecaprevir/pibrentasvir, while the combination of sofosbuvir, velpatasvir, and voxilaprevir is also effective in the rare cases of failure of the initial therapy.

Since 2013, DAA-based therapies exhibited a major clinical benefit, which has become increasingly apparent, provided that HCV eradication in patients with advanced fibrosis/cirrhosis halts the progression from compensated cirrhosis to decompensated cirrhosis, as the number of patients who need liver transplantation due to decompensated cirrhosis has dramatically fallen, i.e., a 48% reduction in the USA [1] and a 60% reduction in Europe [15], when compared to the pre-DAAs era. Even more important, SVR attained through DAA therapy results in a significant reduction in all-cause mortality in patients with advanced fibrosis-hepatitis C [16]. Among 15,059 treated Veterans Affairs patients bearing HCV advanced liver disease (as per FIB-4 index > 3.25), those who achieved SVR had a 79% reduction in mortality, compared to those who did not achieve viral eradication: 2.6 deaths vs. 12.3 deaths per 100 patient-years, respectively, in a mean follow-up of only 1.6 years (*p* < 0.001) [16]. Likewise, a significant decrease in mortality is also present in patients without advanced liver disease who achieve HCV eradication by DAA treatment compared to those who do not obtain SVR [17].

The incidence of HCC decreases significantly after the cure of hepatitis C, either interferon-based [18] or DAA-based treatment [16,19,20]. However, HCC risk is not abolished and “de novo” HCC is regularly diagnosed, even many years after viral eradication. The mechanisms involved in hepatocarcinogenesis following SVR in patients with chronic hepatitis C have not been completely elucidated. It is well known that there is a direct relationship between the fibrosis stage and the occurrence of HCC, being the presence of cirrhosis as the highest risk factor. The long-term duration of hepatic necroinflammatory activity is classically invoked, i.e., repeated cycles of cell death and regeneration lead to genomic instability and the loss of cell cycle control [21]. Epigenetic and gene expression alterations have been demonstrated in the liver tissue of hepatitis C patients, changes which persist after SVR [22]. The long-term duration of HCV infection and the resulting accumulation of hepatic fibrosis are both factors that influence the possibility of HCC, even after viral eradication.

Liver elastography allows for non-invasive, reliable monitoring of liver fibrosis. In Facciorusso’s study [23], significant fibrosis regression was shown after therapy in hepatitis C responder patients undergoing antiviral treatment from 12.3 kPa (9–17.8) to 6.6 kPa (5.3–7.4), while non-responders showed increasing liver stiffness over time, up to 23.7 kPa (15–32.5) at 5 years. Moreover, the proportion of cirrhotic patients decreased by 50% at 6 months and continued to decrease thereafter among the cured patients [23].

The fact that responders and non-responders both continue to generate HCC, albeit in different incidence rates, eloquently shows the multi-factorial nature of post-cure HCC.

A recently published decision–analytical model study calculated that incidence of HCC in individuals with cured chronic hepatitis C would increase from 5.3% in 2012 to 45.8%, peaking in 2040, given that most patients with advanced fibrosis/cirrhosis are currently being treated and their HCV infection is eradicated at present [24]. Guidelines from the main scientific societies recommend that patients with HCV-related cirrhosis should continue surveillance for HCC after viral eradication, but there are still differences among them regarding patients with bridging fibrosis (METAVIR F3). The last *Practice Guidance on the Prevention, Diagnosis, and Treatment of Hepatocellular Carcinoma* by the American Association for the Study of Liver Diseases states that “HCC incidence is significantly lower in post-SVR patients without cirrhosis, and surveillance is not cost-effective or recommended in this population” [25]. In contrast, the *Clinical Practice Guidelines* by the European Association for the Study of the Liver (EASL) published in 2018 claimed that “patients with chronic hepatitis C and bridging fibrosis in the absence of cirrhosis (i.e., METAVIR F3) carry the risk of being under-staged, and thus, at significant risk of HCC. In other words, since the transition from advanced fibrosis to cirrhosis cannot be accurately or reliably defined in all cases, EASL recommends HCC surveillance for patients with bridging fibrosis” [26]. However, in the new EASL *Guideline* presented online on 16 December 2024, the expert panel textually recommend “patients with chronic liver disease and advanced fibrosis without cirrhosis have a higher risk of HCC than the general population, but HCC surveillance cannot currently be recommended in this group owing to insufficient evidence” [27].

The task in the time to come is to clinically identify factors which lead to increased risk of HCC development after SVR and act consequently, creating an ad hoc surveillance of HCC adjusted to the risks for the population with cured HCV. The present work leads to the concept of a reappraisal of current surveillance based on the weight of identified risk factors.

Zangneh et al. developed a Markov model to evaluate the cost-effectiveness of a twice-a-year ultrasound (US) surveillance versus no surveillance in fifty-year-old patients with advanced fibrosis, after HCV eradication. They concluded that HCC surveillance after HCV cure was cost-effective for patients with cirrhosis (approximately USD 43,000 per quality-adjusted life-years after treatment with DAAs) but not for patients with F3 fibrosis [28]. Many variables are considered in these models, including the sensitivity and specificity of abdominal US in the surveillance setting, sensitivity and specificity of abdominal MRI in the diagnosis, and the cost of treatments, such as liver transplantation, but the main driver of the cost-effectiveness of HCC surveillance is the annual incidence of HCC. Previous estimations suggested that surveillance is cost-effective if the HCC incidence rate is greater than 1.5% per year. Zangneh et al. suggested that the threshold for surveillance to be cost-effective after SVR is an annual incidence of approximately 1.32% per year [28].

Accuracy in the assessment of liver fibrosis in patients with hepatitis C is a complex issue. While liver biopsy remains the gold standard, it is not widely available because of its high cost, invasiveness, risks, and the necessity of an expert histological interpretation. Non-invasive tests are increasingly being used for fibrosis staging in chronic hepatitis C. Transient elastography (FibroScan^®^, Echosens, Paris, France) is the most validated one, although it requires expensive equipment not widely available in low-to-middle income countries. Among the blood tests for non-invasive assessment of liver fibrosis, the Enhanced Liver Fibrosis (ELF) test is considered to be a “direct” marker of liver fibrosis since it combines the evaluation of three different products involved in extracellular matrix breakdown, namely hyaluronic acid, N-terminal pro-peptide of collagen III, and tissue inhibitor of metalloproteinase-1. The ELF index has been shown to predict liver fibrosis better than indirect markers in both monoinfected HCV as well as in HIV/HCV coinfected [29]. In addition, the ELF test predicts clinical outcomes, such as mortality in HIV/HCV coinfected women [30]. However, it is expensive and requires payment, since it is patented, and it is not available in many countries, such as our country, Argentina. The “World Health Organization Guidelines for the Screening, Care and Treatment of persons with chronic hepatitis C infection”, version from 2016 [31] recommends using the non-patented, freely available, non-invasive tests APRI [32] and FIB4 [33] to assess the grade of liver fibrosis before viral eradication, while the Veterans Health Administration in the USA and Canada has used FIB4 in the assessment of hepatic fibrosis in the majority of its patients, following the results of their very large validated clinical experience [34]. Non-invasive tests are very useful to exclude advanced fibrosis when the results are below their lower cut-off, transient elastography shows a good performance in diagnosing cirrhosis [35], and APRI shows high specificity (94%) for cirrhosis when values are ≥2 [31]. However, in the real-world setting, when confronted with an individual patient, it might still be difficult to decide whether to maintain (F4) or stop (F3) surveillance for HCC after HCV eradication based only on non-invasive test results.

In the present work, we summarize the recently published knowledge on variables that help to stratify the risk of HCC development after attaining SVR with HCV treatment. We will focus on interferon-free regimens, as these are DAA therapies, specifically in patients without a history of previously treated HCC, before attaining SVR.

The present work is a comprehensive narrative review of the medical literature focusing on the development of de novo hepatocellular carcinoma after curative HCV treatment employing direct acting antivirals. Our conclusions reflect the findings from the reviewed medical literature in the mentioned area.

This is by no means a systematic review, since we have not applied either the search strategy or the methodology of systematic reviews.

## 2. The Occurrence of Hepatocellular Carcinoma After the Cure of Chronic HCV Infection

Recently, some observational studies have evaluated the incidence rate of HCC after SVR with DAAs in patients with chronic hepatitis C and have analyzed baseline and post-therapy variables that may serve to stratify the risk [34,36,37,38,39,40,41,42,43,44,45,46,47,48,49,50,51,52,53,54,55,56,57,58,59,60,61,62,63,64,65,66,67,68]. Table 1 shows a summary of their main results.

Four of these studies revise the database from Veterans Affairs hospitals in the USA [34,36,37,64], ten multi-center studies report findings from various Italian regions [38,39,40,41,42,44,52,53,56,62], four multi-center or single-center studies show results from Spain [45,48,63,65], ten other multi-center or single-center studies originate in Japan [43,46,47,50,51,57,59,60,66,67], one paper shows the experience from the nationwide German Hepatitis C-Registry [58], and multicenter studies show results, one each, from Russia [49], Israel [54], France [55], China [61], and Egypt [68]. In all, these results reflect the follow-up of more than 220,000 patients treated with DAAs.

The conclusions are necessarily provisional because the median follow-up post-DAA treatment is still rather low, ranging from 24 weeks to 4.84 years. Clinically relevant data are expected in this area in the next few years.

Most of the Italian studies included patients with cirrhosis; one out of the four Spanish studies analyzed patients with compensated advanced chronic liver disease (cACLD), defined by liver stiffness measurement (LSM) ≥ 10 kPa, devoid of prior hepatic decompensation (i.e., ascites, variceal bleeding, hepatic encephalopathy, or jaundice)), while the remaining three included patients either with cirrhosis or advanced fibrosis. Regarding the series from Veterans’ hospitals in the USA, all of them evaluated patients within the entire fibrosis spectrum. Within their population, the prevalence of cirrhosis ranged from 20% to 38% [34,36,37,64]. The overall annual incidence rate of HCC in patients with cirrhosis ranged from 1.49% [40] to 5.0% [62] in the first year post-SVR.

As already mentioned, follow-up after SVR is still preliminary for drawing definite conclusions as to whether the incidence of HCC will effectively decrease over time. Romano, from the northern Italian Venetian region, found that the HCC incidence rate significantly declined among patients with Child–Pugh A cirrhosis, from 1.49/100 patient-years (95% CI, 1.03–2.08) in the first year to 0.20/100 patient-years (95% CI, 0.05–0.51) in the second year post-DAA treatment [40]. In the study by Ioannou, the annual HCC incidence appeared to decrease over time through the first four years after DAA treatment (in patients with a baseline FIB-4 ≥ 3.25, from 3.8% at the first year to 2.4% at the fourth year) [34]. In another recently published retrospective study based on the Veterans Affairs database, the HCC annual incidence rate seemed to decline significantly among patients with cirrhosis up to six years after SVR (from 2.71/100 person-years for those who had accrued only one to two years since SVR to 1.65/100 person-years for those who has accrued >four to six years since SVR) [69]. However, the annual incidence rate of HCC remained stable for patients with cirrhosis with more than six years of follow-up post HCV eradication (1.68/100 person-years) [69]. This last finding would support the proposal of life-long surveillance of HCC in that subgroup of patients with cured HCV.

### 2.1. Demographic Characteristics of the Patients Studied and Their Association with the Risk of HCC Occurrence

Regarding patient demographics, the median age of patients was in their sixties in most European studies. In contrast, patients were older in the Japanese studies, with median ages ranging from 65 to 72 years. Approximately 60% of the analyzed individuals were male in the studies from Italy and Spain, and this percentage decreased to 36–48% in the ten studies from Japan. In contrast, the population enrolled in the studies at Veterans hospitals in the US and Canada was almost exclusively male, i.e., 96–97%. This is worth mentioning since the male gender was found to be an independent predictor of increased risk of HCC after SVR in six studies [42,43,44,46,64,68]. Likewise, older age was shown to be an independent predictor of HCC occurrence in seven studies [37,41,44,46,52,55,68]. Male gender and old age are both features to consider in a still-to-come reappraisal of HCC surveillance after HCV eradication.

### 2.2. Other Variables Associated with Increased Risk of HCC Occurrence

Among the baseline variables identified as independent predictors of the occurrence of HCC in the multivariate analyses, there is an important role for non-invasive fibrosis tests. Baseline FIB4 was found to be a significant marker of de novo HCC in five studies [34,42,43,64,65], APRI in one [40], and liver stiffness measurement (LSM) by different elastography devices in seven [42,45,50,52,55,56,65]. In six of these, vibration-controlled transient elastography (VCTE; FibroScan^®^) was used and, in the other, acoustic radiation force impulse (ARFI) elastography [50] was used. Furthermore, the results of these non-invasive fibrosis markers were also found to be predictors of increased risk of HCC during follow-up after HCV eradication: FIB4 in six studies [37,45,49,59,61,67] (absolute value in four, Δ FIB4 in two); LSM in four studies [45,48,51,53] (absolute value in two, ΔLSM in two), in three of them, by FibroScan^®^, and in the other by magnetic resonance elastography; and APRI in one other study [37]. The results reported by Ioannou et al. are eloquent concerning the potential usefulness that a freely available marker, such as FIB4, can have in predicting the incidence of HCC after SVR [34]. Among their patients with cirrhosis, those with a FIB4 ≥ 3.25 and <3.25 show completely divergent results. The former showed an annual HCC incidence of 3.66/100 patient-years, and the latter had a significantly lower incidence of HCC, 1.16/100 patient-years, (adjusted HR 2.14, 95%CI, 1.66–2.75) [34]. Even among patients without cirrhosis, there was a statistically significant difference in the occurrence of HCC post-SVR among those with a baseline FIB4 ≥ 3.25 (annual incidence, 1.22/100 patient-years) versus those with a baseline FIB4 < 3.25 (annual incidence, 0.24/100 patient-years) (adjusted HR 3.56, 95%CI, 2.74–4.53) [34].

Several studies [70,71,72], and even a systematic review with meta-analysis [73], have shown the association of liver elastography findings with an increased risk of HCC, cirrhosis decompensation, and even liver-related death in patients with chronic hepatitis C before antiviral treatment. Several other studies have shown the usefulness of elastography as a predictor of HCC after viral eradication. A systematic review and meta-analysis identified eight studies that analyzed the performance of LSM as a predictor of HCC occurrence after achieving SVR, including 3398 patients [73]. The pooled hazard ratio for HCC occurrence determined by LSM was 3.43 [95% CI, 1.63–7.19] for patients whose elastography result was above the cut-off proposed by a ROC curve, compared to patients with a value below that cut-off, indicating that LSM might help predict HCC occurrence [73]. Unfortunately, there are still discrepancies about the best cut-off value to indicate an increased risk of HCC, or even if it is better to define it with a baseline elastography or one performed after viral eradication. Table 2 shows the results of studies that evaluate different types of elastography in their ability to predict the occurrence of HCC after HCV eradication.

One parameter related to hepatocellular function, such as serum albumin (pre-DAA treatment), was a proven independent predictor of HCC occurrence in nine studies [39,43,44,45,48,59,60,64,68]. Signs of portal hypertension, such as thrombocytopenia or the presence of esophageal varices, were both found to be predictors of de novo HCC after SVR in seven studies (thrombocytopenia in five [39,52,54,59,62], presence of varices in two [39,62]). Combining these two variables has proven very useful to stratify the risk of HCC after the HCV cure. In the study by Calvaruso et al., carried out in patients with cirrhosis, those who had a baseline serum albumin < 3.5 g/dL and a platelet count < 120,000/µL (the two variables found as predictors in their multivariate analysis) had an annual incidence of HCC of 5.6% versus only 0.8% in those who had pre-treatment serum albumin and platelet count above the mentioned cut-off values [39].

Regarding other analyzed variables after HCV eradication following successful antiviral therapy, seven studies (all of them from Japan) showed that alpha-fetoprotein (AFP) levels were independent predictors of increased risk of HCC occurrence [43,46,51,59,60,66,67]. The best cut-off values were 5, 6, or 7 ng/mL in the different studies, which strongly point out that close attention should be paid to patients who show slightly elevated AFP levels after hepatitis C eradication. In the study by Watanabe, patients with a baseline FIB4 > 4 and post-SVR AFP > 6 ng/dL had one and two-year cumulative HCC incidence rates of 6.1% and 14.4%, respectively, compared with two-year incidence rates of only 0.4% in those with a baseline FIB4 < 4 and post-SVR AFP < 6 ng/dL (*p* < 0.001) [43].

### 2.3. Are There Variables That Allow Us to Identify Patients with a Lower Risk of HCC Occurrence?

When reviewing the variables that are independent predictors of a higher risk of HCC, an interesting objective would be to find a subgroup of patients whose risk of tumor occurrence is negligible. In the recently published study by Alonso, who analyzed 993 patients with compensated advanced fibrosis (mean baseline LSM of 19.9 kPa) during a median follow-up of 45 months, the author proposed a transient elastography-based HCC-risk model [45]. Briefly, the score ranged from 0 adding up to 3, according to whether or not the patient presents the following variables, all of which were found to be significant predictors of HCC development, according to the multivariate analysis: (a) a baseline LSM > 17.3 kPa, (b) a baseline serum albumin < 4.2 g/dL, and (c) a one-year follow-up after DAA treatment delta LSM < 25.5% (a decrease in LSM smaller than 25.5% compared to pre-therapy). In patients with a score of 1–3 (at least one of the variables was present), the HCC occurrence at three years was 5.2%, while in patients with a score of 0 (none of the 3 variables was present), the incidence was 0% [45]. Thus, this Spanish study described a subgroup of patients without any incidence of HCC, at least in the three-year observation period. This interesting finding is worth being validated by other groups with a longer follow-up, as it is targeting a subset of patients in whom HCC surveillance could at least potentially be discontinued. Overall, it is an eloquent example of heterogeneity affecting the incidence of HCC post-HCV cure. If confirmed, this information might be potentially applicable in future reappraisals of HCC surveillance and its cost-effective approach.

As already mentioned, patients with F3 fibrosis have a lower incidence of HCC than patients with cirrhosis. Another Spanish multicenter study, recently published, evaluated the risk of HCC occurrence after SVR in 506 patients with well-defined F3 fibrosis treated with direct-acting antivirals (DAAs) [74]. Stage 3 fibrosis was defined by a two-step procedure: a. Patients need to have transient elastography values of 9.5–14.5 kPa. b. There is an exclusion of nodular liver surface, splenomegaly, ascites, or collaterals on imaging, thrombopenia, or esophagogastric varices [74]. In a median follow-up of 33.7 months, five patients developed HCC and one developed intrahepatic cholangiocarcinoma. The primary liver cancer incidence rate was 0.49/100 patient-years (95% CI 0.2–1.01). In the multivariate analysis, only a combined variable, males older than 55 years, was significantly associated with a higher risk of primary liver tumor (HR 7.16; 95% CI 1.23–41.75; *p* = 0.029) [74]. In that subset, the incidence rate was 1.10/100 patient-years (95% CI 0.30–2.81). This study delineated another subgroup for which surveillance could be less stringent: young, female patients with well-defined F3 fibrosis and cured hepatitis C. In addition, in a prospective, single-center study performed by the BCLC group in Barcelona, Spain, 185 patients (63 in the F3 stage, 122 with cirrhosis) had a median follow-up of 52.4 months after HCV eradication. The HCC incidence was 2.24/100 person-years (95% CI 1.21–4.17) among patients with cirrhosis, but not a single HCC was diagnosed among the F3 [63]. More studies with a larger number of patients with F3 fibrosis and longer follow-up are necessary, but current evidence describes a fairly low incidence of HCC after the cure of hepatitis C. At present, discussion is open as to whether or not these patients should be enrolled in a surveillance protocol for HCC or whether they should undergo a different regimen adapted to their lower risk.

Another tool for predicting the risk of HCC that can be used, especially in patients without precise information of their liver disease stage before DAA treatment, is the recently described aMAP risk score [75]. To develop this score, more than 17.000 patients were studied, comprising approximately 10.000 treated Asian patients with chronic hepatitis B (CHB), 2.500 treated Caucasian patients with CHB, 3.500 treated patients with HCV, and 720 patients with non-viral chronic hepatitis from eleven international prospective observational cohorts or randomized controlled trials. Patients were divided into training cohorts and nine validation cohorts (with different etiologies and ethnicities). The aMAP risk score involves *age*, *male gender*, *albumin*, *bilirubin*, and *platelet count,* and may range from 0 to 100. Patients with a score < 50 had three- and five-year cumulative incidences of HCC of 0 and 0.8% (with a negative predictive value of 99.3–100%). Patients with a score > 60 had three- and five-year cumulative incidences of HCC of 8.1 and 19.9% (with a positive predictive value of 6.6–15.7%). One strength of the aMAP score is its validation in Asian and Western cohorts with hepatitis B and C. Patients with aMAP score < 50 accounted for 44% of the overall population, with a very low HCC incidence (<0.2% per year) [75].

### 2.4. A Second Cause of Chronic Liver Disease: The Risk of HCC Due to a Concurrent Cause of Liver Disease

Another aspect to take into account, which might impede the decrease in the risk of HCC development, despite hepatitis C cure, is the presence of a concomitant or simultaneous second cause of chronic liver disease, such as alcohol intake or metabolic dysfunction-associated fatty liver disease (MASLD). The clinical situation involving more than one etiological factor is relatively frequent today due to the growing epidemic of overweight/obesity and type 2 diabetes worldwide. The presence of type 2 diabetes before HCV treatment was shown to be an independent predictor of HCC development after cure in three of the studies reviewed [42,55,59].

The relationships of the hepatitis C virus, insulin resistance, and type 2 diabetes are intriguing and have stimulated much research a few years ago. First, it was postulated that HCV infection itself might cause insulin resistance, altering the insulin signaling cascade. Several case–control studies had shown that the prevalence of type 2 diabetes was significantly higher in patients with chronic hepatitis C than in controls. In addition, two prospective community-based studies from Taiwan, comparing patients with chronic hepatitis C and controls without diabetes at baseline, showed that HCV infection was an independent predictor of incident diabetes [adjusted HR 1.63 (95% CI, 1.31–2.02) in the study by Lin] [76], [HR 1.7 (95% CI, 1.3–2.1) in the study by Wang] [77]. Finally, after receiving interferon-based antiviral treatment, a two-thirds reduction in the incidence of diabetes was observed in patients with viral eradication compared to those who had not been cured in a study from Japan [78]. However, the causes of insulin resistance in patients with type 2 diabetes include genetics, overnutrition, a Western diet rich in fructose, overweight/obesity, and a sedentary lifestyle, and hepatitis C may have only been a cofactor. Approximately 65% of patients with type 2 diabetes develop MASLD [79], which could be the factor that prevents the regression of fibrosis in patients with cured hepatitis C or that drives progression of the liver disease.

To date, there are few studies published on the evolution of patients with coexisting MASLD or diabetes after HCV eradication. The implication of the multiple etiology was evaluated in a single-center study in Japan by Ogawa [47], who, among the analyzed variables, included the FAST score. The latter combines information of LSM and controlled attenuation parameter (CAP), both by FibroScan^®^ and AST, in one formula and it is employed to non-invasively identify patients for clinical trials who are at risk of progressive nonalcoholic steatohepatitis with significant fibrosis [80]. Ogawa and colleagues found that a FAST score ≥ 0.35 at 12 weeks after completing a DAA treatment was associated independently with the development of HCC (adjusted HR 4.42: 95% CI 1.02–19.9; *p* = 0.043) [47]. Furthermore, in another study from China, both the presence of diabetes (HR 4.2; 95% CI 2.4–7.4) and MASLD (HR 2.4; 95% CI 1.3–4.2) were found to be associated with an increased incidence of HCC after HCV eradication [81]. MASLD was present in 27.1% of the 484 patients who received treatment with DAAs, and in 62.1% of the 29 patients who developed HCC during follow-up [81]. Perhaps the most provocative findings are those recently published by Liu et al. from Taiwan, who studied 1598 patients after the curing of hepatitis C, 919 and 679 with and without MASLD, respectively, as determined by CAP evaluation and the presence of metabolic features [82]. The cumulative HCC incidence was significantly higher in patients with MASLD than in those without MASLD (2.41 and 0.76 per 100 person-years of follow-up, respectively). Multivariable analysis showed that MASLD was independently associated with HCC (adjusted hazard ratio 2.07; 95% CI 1.36–3.16), in addition to age, sex, LSM, platelet count, and AFP [82].

Furthermore, some drugs commonly prescribed for diabetes or obesity might also modify the incidence of HCC. Although metformin’s effect on MASH histology has not yet been properly evaluated, importantly, in a retrospective study concerning patients with diabetes bearing advanced liver fibrosis or cirrhosis, metformin use was associated with a lower risk of HCC (sHR: 0.25; 95% CI: 0.11–0.58, *p* = 0.001), after propensity-score and covariate-adjusted analyses [83].

Likewise, glucagon-like peptide-1 receptor agonists (GLP1-RAs), approved for type 2 diabetes and obesity, achieve weight loss ≥ 5% from baseline in 60%, 85%, and 90% of patients treated with liraglutide, semaglutide, and tirzepatide, respectively. Semaglutide has been shown to be superior to a placebo in achieving both the resolution of steatohepatitis with no worsening of liver fibrosis and improvement in liver fibrosis without worsening of steatohepatitis in the phase 3, randomized controlled, not-yet-published ESSENCE trial [84]. In part 2 of the study, the aim will be to demonstrate that semaglutide 2.4 mg weekly reduces liver-related clinical events at week 240 compared to a placebo (results expected in 2029). Meanwhile, several retrospective cohort studies have compared the incidence of clinical outcomes, such as cirrhosis complications or HCC, in patients with type 2 diabetes who started treatment with GLP-1 RA versus those under other hypoglycemic therapies. In one of such retrospective studies, including 1.890.020 diabetic patients, among those with a prior diagnosis of MASLD or MASH, GLP-1 RAs were associated with a significantly lower risk of developing HCC compared to patients under insulin (HR, 0.34; 95% CI, 0.17–0.70), but not compared with other anti-diabetic medications [85]. In another cohort study that included 1.651.452 patients with diabetes who were prescribed GLP-1 RAs, insulin, or metformin, GLP-1 RA use was associated with a significant risk reduction in 10 obesity-associated-cancers, including HCC (HR, 0.47; 95%CI, 0.36–0.61), compared to insulin use. The reduction in HCC incidence was attained with GLP-1 RAs or metformin [86].

Finally, patients with diabetes or obesity frequently have atherogenic dyslipidemia for which they receive statins. Various meta-analyses have found that statin use was associated with a reduced risk of HCC occurrence (RR: 0.54, 95% CI: 0.47–0.61) compared with nonusers. A decrease in rate of HCC was also significant among patients with diabetes (RR: 0.44, 95% CI: 0.28–0.70) or cirrhosis (RR: 0.36, 95% CI: 0.30–0.42) [87]. These results evidence the complexity of the problem of a second liver disease in patients with HCV eradication. While diabetes or MASLD can drive a greater progression of liver fibrosis and risk of HCC, some therapies, such as metformin, GLP-1 Ras, or statins, might decrease the risk of HCC.

**Figure 1:**
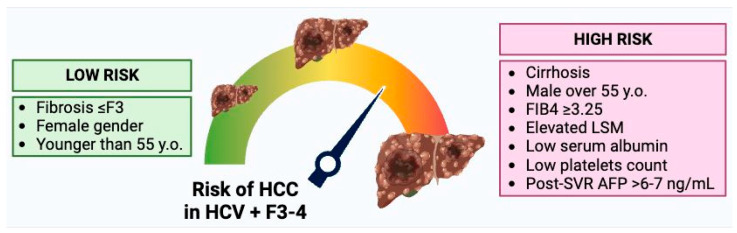
Factors that can influence the risk of developing de novo HCC after HCV eradication by direct-acting antiviral therapy. AFP, alpha-fetoprotein; HCC, hepatocellular carcinoma; LSM, liver stiffness measurement; SVR, sustained virological response; y.o., years old.

According to all this described information, it becomes evident that it will be unlikely that a single variable will allow us to define which patient remains at increased risk of HCC after achieving SVR. A very recent European multicenter study tried to develop algorithms for risk stratification in a derivation cohort of 475 patients with compensated advanced chronic liver disease (cACLD) and SVR with DAA therapy, then validated their results in 1500 patients with cACLD and HCV eradication from other European centers [88]. During a median follow-up of 41 months, 22/475 patients with cACLD (4.6%, 1.45/100 patient-years) developed de novo HCC. After significant univariable associations with HCC occurrence were found, multivariable models were built. The models comprising age ≥ 59 years, follow-up AFP ≥ 4.6 ng/mL, follow-up LSM ≥ 19 kPa, and follow-up albumin < 42.0 g/L, with and without alcohol consumption above the threshold, showed the highest predictive ability. A simple score was derived from adjusted hazard ratios assigning three points for follow-up (FU) AFP ≥ 4.6 ng, two points for age ≥ 59 years, two points for alcohol consumption above the threshold, one point for FU-LSM ≥ 19 kPa, and one point for FU albumin < 42 g/L. Patients were then stratified into low-risk (0–3 points, *n* = 308) and high-risk (4–9 points, *n* = 160) groups. The low-risk group comprised 66% of the derivation cohort and the high-risk group included the remaining 34%. This dichotomization identified very effectively patients at very low risk of HCC at four years versus substantial risk of HCC at four years: 0% vs. 16.5% [88]. Stratification also accurately separated low-risk and high-risk groups in the validation cohort. It is interesting to mention that approximately two-thirds of patients were identified as having an HCC risk of <1%/year [88]. Figure 1 shows the complex balance between factors that can increase or decrease the risk of developing de novo HCC.

## 3. Risk-Based Surveillance Strategies for HCC

One of the main problems with HCC surveillance is that we tend to use the same “one-size-fits-all” approach for patients who might be at different relative risks of developing HCC. As we have reviewed, the annual incidence of HCC in patients with HCV eradication (and without second liver disease) can range from <0.49% in women with F3 fibrosis [74] to approximately 5% in patients with cirrhosis and FIB4 > 3.25, both pre- and post-DAA treatment [34]. The surveillance strategy that is currently recommended (twice a year abdominal ultrasonography with or without serum AFP) comprises two readily available, inexpensive tests, which should be easy to perform. However, real-life studies regrettably show that adherence to its simple schedule is as low as 17% (regular surveillance) or 38% (inconsistent surveillance) [89]. The consequence is that our current strategy may be excessive in patients with lower risk of HCC but insufficient in those with higher risk. Patients could and should be separated into those with lower, medium, or higher risk of developing HCC, and, accordingly, indicate different screening tests or intervals between studies. A recent meta-analysis showed that sensitivities of hepatic US alone or with AFP measurement to detect early-stage HCC were 45% and 63%, respectively [90]. The last goal of surveillance for HCC is to increase patient survival, and for this, early-stage tumors must be diagnosed. Most likely, US plus AFP screening is missing a percent of early HCC diagnoses in patients at higher risk for HCC.

Kim et al. compared the HCC detection rate of US and liver-specific contrast-enhanced MRI in 407 patients with cirrhosis and an estimated annual risk of HCC greater than 5% [91]. HCC was diagnosed during the study in 43 patients. They found that the HCC detection rate of MRI was 86.0% (37/43), significantly higher than the 27.9% (12/43) of US (*p* < 0.001). Most of the patients with HCC received curative treatments and their three-year survival rate was not inferior to those without HCC [91]. The problem when using full-procedure MRI as a screening test for HCC is that it is expensive and time-consuming. For this reason, different kinds of abbreviated MRI (AMRI) protocols have recently been proposed for HCC surveillance [92,93,94,95]. AMRI involves performing a limited number of sequences but maintaining a high detection rate of focal liver lesions. The main protocols investigated have been non-contrast AMRI (NC-AMRI), including T1-weighted, T2-weighted, and DWI sequences [93], or with intravenous contrast administration, either with dynamic extra-cellular contrast-enhanced AMRI (DEC-AMRI) [94] or with hepatobiliary phase contrast-enhanced (HBP-AMRI) [95]. All the approaches can be performed in about ten minutes and show a higher sensitivity and accuracy than US [92]. Multiple published studies have reported promising results with AMRI. However, it should be clarified that, to date, most of the studies have been retrospective and simulated, reviewing sequences of patients who had been studied with a complete MRI. Some prospective studies are at present ongoing, comparing different protocols of AMRI with abdominal ultrasound for HCC surveillance. In a recently published prospective paper, Kim et al. compared the diagnostic performance of annual NC-AMRI versus biannual US as HCC surveillance modalities in 208 high-risk patients during a follow-up of 30 months, among whom, 34 were diagnosed with HCC [96]. The sensitivity of annual NC-AMRI (71.0%) was marginally higher than that of biannual US (45.2%) (*p* = 0.077). A simulation of alternating US and NC-AMRI at six-month intervals yielded a sensitivity of 83.9%, significantly exceeding that of biannual US (*p* = 0.006) [96]. This may be the best surveillance strategy for high-risk patients. A study evaluating the cost-effectiveness of risk-stratified HCC surveillance strategies in patients with cirrhosis concluded that AMRI for high- and intermediate-risk patients had the lowest incremental cost-effectiveness ratio (USD 2100 per quality-adjusted life year gained) and was cost-effective [97].

Regarding the screening tests that we usually use in surveillance for HCC, another aspect to discuss is that AFP measurement will probably have a better performance in patients with cured hepatitis C, particularly with an increase in its specificity. Patients with chronic hepatitis B or C and ongoing necroinflammatory activity may have fluctuating levels of AFP, as a reflection of the phenomena of necrosis and cell regeneration. This produces false-positive results and an important increase in the costs of surveillance because, when a patient shows a normal US plus AFP > 20 ng/mL, a contrast-enhanced computed tomography scan or MRI should be performed. In contrast, after HCV eradication, when hepatocyte necrosis subsides, AFP levels are expected to be fully normal. This has already been demonstrated in patients with chronic hepatitis B, firstly in Asian studies [98] and then in Europe [99]. The Milan group evaluated HCC surveillance by semiannual ultrasound evaluation and serum AFP determination in 258 patients with HBV-compensated cirrhosis who were receiving antivirals and had normal aminotransferases and normal serum AFP levels (≤7 ng/mL) at baseline. During a median period of 96 months of antiviral therapy, 3.947 AFP values were collected, and the median AFP level was 2 ng/mL. Among 12 patients who experienced an AFP rise > 7 ng/mL, 11 developed an HCC and 1 had liver metastases to lung cancer [99]. An AFP > 7 ng/mL had a 99.6% specificity and 91.7% positive predictive value for HCC, although sensitivity was 31.4% [99]. Similar results are expected in patients with cured hepatitis C. To improve sensitivity, a lower cut-off of AFP (around 7 ng/mL), instead of the presently proposed 20 ng/mL, should be tested in future studies.

## 4. Conclusions

The present work reviews the emergence of HCC occurring after the successful removal of its main etiological factor, namely HCV infection, through DAA therapy. Our purpose is to alert acting physicians regarding the persistence of the risk of developing de novo HCC, following HCV eradication. Therefore, there is a need to maintain an HCC surveillance strategy looking for the emergence of de novo tumors post-DAA successful treatment.

It is important to emphasize that our proposal refers to patients with cured HCV with no prior history of primary liver cancer. Certainly, the vast majority of patients with hepatitis C who receive therapy with DAAs belong to this group. Our proposal does not apply to patients with hepatitis C with a previous history of HCC having received curative interventions for their HCC tumor. These are far less frequent but do have a significantly higher HCC recurrence rate. Indeed, Conti et al. compared HCC detection in 17 out of 59 patients (28.81%, 95% CI: 17.76–42.07) in patients with cured HCV who had previous HCC versus 9 out of 285 patients (3.16%, 95% CI: 1.45–5.90) without previous HCC [38], after a 24 week follow up. Thus, these are two clearly different scenarios.

Based on our review, it is clear that there is a considerable amount of relevant medical information that we should always bear in mind when managing patients with chronic hepatitis C in the process of receiving DAA therapy. In the first place, we should obtain the most accurate information regarding their liver fibrosis stage (a very often-neglected aspect in the context of the high expectations of attaining SVR through DAAs). When liver biopsy or transient elastography unquestionably show the absence of fibrosis or mild fibrosis, patients can be safely discharged without any need of implementing HCC surveillance. On the contrary, if patients have a transient elastography or biopsy reliably showing F3 or F4 fibrosis, it would still be helpful to know the FIB4 result before and after treatment, since the latter score adds significant information on HCC risk after cure. Even among patients with a “clinical” diagnosis of cirrhosis (signs of portal hypertension, esophageal varices, thrombocytopenia, collateral circulation, etc.), it would still be useful to have their FIB4 and elastography values, before and after treatment, since both help to stratify the risk of HCC. In addition, when dealing with patients bearing advanced fibrosis/cirrhosis, it is very useful to determine AFP values after DAA therapy (probably best at the twelve-week post-treatment control). If these results exceed 6–7 ng/mL, this could indicate an increased risk of developing HCC, according to the most recent information.

If a former patient with hepatitis C is referred to us after DAA treatment with accomplished viral eradication and we lack information regarding liver fibrosis, the aMAP risk score would be useful to stratify their risk of HCC.

In Table 3 we attempt to classify patients with advanced fibrosis/cirrhosis into groups with low, medium, or high risk of HCC, according to the presence or absence of variables mentioned in this review. In addition, we have associated each group with proposals for different HCC surveillance strategies.

We will continue to learn how to stratify the risk of HCC in patients with cured hepatitis C, alongside a more extended observation period during post-treatment follow-up. More studies are needed in many respects. Among them, a very important issue is as to whether the regression/remodeling of liver fibrosis ensuing from hepatitis C eradication is associated with the reversal of the hepatocarcinogenesis process.

In the particular setting of DAA-cured hepatitis C, the clinical and scientific basis for a reappraisal of HCC surveillance methodology is underway. This work offers recent available data, serving as just a starting point.

We should prepare to deal with the coming epidemic of treated (and cured) patients with hepatitis C, and the best way to do so is to better understand and measure the impact of the post-hepatitis C variable degrees of advanced fibrosis, portal hypertension, coexistence with other etiological factors, namely MASLD, or drug-induced liver disease, and be able to generate a cost-effective approach for HCC screening in this setting.

## Figures and Tables

**Table 1:** Studies evaluating the incidence rate of hepatocellular carcinoma (HCC) in patients with chronic hepatitis C after viral eradication with direct-acting antivirals (DAAs), including the baseline or post-sustained virologic response (SVR) variables, with a statistically significant association to an increased risk of HCC occurrence, according to the multivariate analysis.

Study (Year; Reference)	*N*	Age (Years)/Males	Cirrhosis (%)	Follow-Up	Annual Incidence of HCC	Significant Baseline Variables [Adjusted HR (95% CI)]		Significant Post-SVR Variables [Adjusted HR (95% CI)]	
Ioannou et al., 2019; [34]	29,033	62/97%	20	3.0 years	2.2%	FIB4 > 3.25	2.14 (1.66–2.75)		
Kanwal et al., 2020; [37]	18,076	62/96%	38	2.9 years	2.2%	Age Cirrhosis Genotype 3 Alcohol abuse	1.03 (1.02–1.05) 4.13 (3.34–5.11) 1.60 (1.08–2.38) 1.24 (1.03–1.50)	Changes in FIB4	6.99 (4.98–9.81) ^a^
Conti et al., 2016; [38]	285	63/60%	100	24 weeks	NA	Child–Pugh	4.18 (1.17–14.8)		
Calvaruso et al., 2018; [39]	2249	65/57%	100	14 months	2.6%	Albumin < 3.5 g/dL Platelets < 120 × 10^9^/L	1.77 (1.12–2.82) 3.89 (2.11–7.15)		
Romano et al., 2018; [40]	3917	58/62%	70	75 weeks	1.49%	APRI ≥ 2.5 HBsAg+	2.03 (1.14–3.61) 3.99 (1.24–12.91)		
Lleo et al., 2019; [41]	1766	62/62%	100	1 year	2.4%	Age > 50 years Esophageal varices	4.36 (1.04–18.3) ^b^ 4.97 (1.55–16.0) ^c^		
Degasperi et al., 2019; [42]	505	64/60%	100	25 months	3.4%	Male gender Diabetes LSM > 30 kPa FIB4	6.17 (1.44–26.47) 2.52 (1.08–5.87) 1.03 (1.01–1.06) 1.08 (1.01–1.14)		
Watanabe et al., 2019; [43]	1174	65/46%	NA	77 weeks	1.9%	Male gender Serum albumin FIB4 > 4.0	2.46 (1.007–6.026) 0.229 (0.087–0.602) 1.069 (1.024–1.115)	AFP > 6 ng/mL	1.11 (1.054–1.172)
Quaranta et al., 2020; [44]	1202	64/58%	100	25 months		Age Male gender Serum albumin Genotype 3 Anti-HBc	1.08 (1.04–1.13) 2.76 (1.28–5.96) 3.94 (1.81–8.58) 5.05 (1.75–14.57) 1.99 (1.01–3.95)		
Alonso et al., 2020; [45]	993	62/56%	100	45 months	1.4%	Serum albumin LSM > 17.3 kPa	0.400 (0.206–0.766) ^d^ 1.036 (1.015–1.057)	1-year ΔLSM 1-year FIB4	0.988 (0.982–0.994) ^e^ 1.069 (1.001–1.142)
Ogawa et al., 2020; [46]	1670	67/43%	26	3.5 years		Age > 70 years Male gender Cirrhosis	2.39 (1.41–4.06) 2.07 (1.23–3.48) 2.97 (1.78–4.92)	Albumin < 3.5 g/dL AFP > 7	2.72 (1.39–5.33) 4.92 (2.72–8.88)
Pons et al., 2020; [48]	572	64/49%	cACLD	2.9 years	1.5%	Albumin < 4 g/dL	3.27 (1.45–7.36)	Albumin < 4.4 g/dL LSM > 20 kPa	2.36 (1.02–5.47) 4.53 (1.36–15.08) ^f^
Nabatchikova et al., 2020; [49]	229	54/49%	100	30 months	3.1%	Bilirubin > 34 µmol/L	3.42 (1.17–9.97)	FIB4 > 3.25	15.15 (1.94–118.18)
Tachi et al., 2017; [50]	233	71/46%	19	18 months	2.3%	LSM ≥ 1.73 m/s ^g^	8.35 (1.62–43.09)		
Tamaki et al., 2019; [51]	346	68/36%	11	26 months	NA			Age AFP > 6.5 LSM ≥ 3.75 kPa ^h^	1.72 (1.06–2.78) 2.7 (1.15–6.37) 3.51 (1.24–9.99)
Rinaldi et al., 2019; [52]	258	68/55%	100	NA	NA	Age Platelet count LSM ≥ 27.8 kPa	1.067 (1.010–1.127) NA for platelet count 1.113 (1.024–1.210)		
Ravaioli et al., 2018; [53]	139	62/65%	100	15 months	NA	Child–Pugh B	4.046 (1.542–10.618) ^i^	Δ LSM < 30%	5.360 (1.561–18.405) ^j^
Peleg et al., 2019; [54]	515	54/54%	77	24 months	2.61%	Liver steatosis Platelet count	7.51 (3.61–13.36) 0.98 (0.97–0.99)		
Shili et al., 2018; [55]	799	61/52%	NA	5 months	NA	Age Diabetes LSM	1.05 (1.02–1.09) per year 3.03 (1.46–6.28) 1.05 (1.03–1.06) per kPa		
Morisco et al., 2021; [56]	687	64/54%	100	24 months	1.6%	LSM >20 kPa	7.2 (1.9–26.7)		
Murakawa et al., 2024; [57]	1001	68/45%	NA	1338 days	NA			Elevated GGT	2.38 (1.1–5.17)
Tacke et al., 2022; [58]	6982	53/61%	33	NA	NA			Elevated GGT	OR 3.12 (1.82–5.33)
Abe et al., 2020; [59]	880	66/48%	0	43 months	0.7%	Albumin < 3.95 g/dL	3.57 (1.35–9.95)	FIB4 > 3.25 AFP > 6 ng/mL	6.01 (1.60–26.94) 5.70 (1.47–19.34)
Abe et al., 2020; [59]	188	70/48%	100	43 months	2.6%	Score ALBI > −2.3 Diabetes Platelets < 82 × 10^9^/L	4.26 (1.70–11.15) 3.80 (1.35–10.65) 4.14 (1.55–11.20)	Score ALBI > −2.3	5.42 (1.59–17.06)
Ogata et al., 2017; [60]	1065	67/42%	100	1.3 years		Albumin < 3.8 g/dL AFP > 5 ng/mL	3,95 (1.12–13.9) 12.6 (1.66–96.0)	AFP > 5 ng/mL	12.1 (CI are NA)
Zou et al., 2022; [61]	701	57/24%	14	4.84 years	0.28%			FIB4 > 3.25	3.14 (1.40–7.05)
Gardini et al., 2019; [62]	416	63/58%	100	18 months	5.0%	Score ALBI Platelet count	2.35 (1.05–5.25) 0.92 (0.85–1.0)		
Sanduzzi et al., 2022; [63]	185	66/52%	66	52 months	1.46% (2.24% in cirrhosis)	NA		NA	
Kramer et al., 2022; [64]	92,567	61/96%	26.5	2.51 years	2.0% ^k^	Male gender Esophageal varices Serum albumin Genotype 3 FIB4 > 3.25 Bilirubin	1.89 (1.37–2.59) 1.73 (1.57–1.97) 0.48 (0.44–0.52) 1.47 (1.27–1.71) 2.49 (2.11–2.94) 1.24 (1.15–1.34)		
Ampuero et al., 2022; [65]	1054	NA	cACLD	49 months	NA	FIB4 > 3.25 LSM Cirrhosis	2.26 (1.08–4.73) 1.02 (1.0–1.04) 3.15 (1.36–7.27)		
Nagata et al., 2017; [66]	669	69/46%	NA	1.8 years	NA			AFP > 5.4 ng/mL WFA + M2BP > 1.8 C.O.I. ^l^	NA
Tamaki et al., 2022; [67]	1325	72/40%	100	2.96 years				FIB4 > 4.28 AFP > 4.0 ng/mL GGT > 28 UI/L	2.33 (1.5–3.7) 1.97 (1.2–3.3) 1.88 (1.2–3.0)
Shiha et al., 2020; [68]	2372	NA	73	24 months	2.33%	Age > 54 years Male gender AFP > 20 ng/mL Albumin < 3.8 g/dL Cirrhosis	1.072 (1.04–1.10) 3.61 (2–6.52) 2.83 (1.55–5.18) 1.86 (1.15–3.0) 3.48 (1.69–7.17)		

HCC, hepatocellular carcinoma. DAAs, direct-acting antivirals. SVR, sustained virological response. HR, hazard ratio. CI, confidence intervals. NA, not available. LSM, liver stiffness measurement. AFP, alpha-fetoprotein. cACLD, compensated advanced chronic liver disease. GGT, gamma glutamyl transpeptidase. ^a^ Unchanged, high risk (baseline and at 12-months post-SVR FIB4 > 3.25) in respect to unchanged, low risk (baseline and at 12-months post-SVR FIB4 < 1.45), ^b^ ≥50 years old in respect to <50 years old. ^c^ F3 varices in respect to no varices. ^d^ Baseline albumin > 4.2 g/dL in respect to <4.2 g/dL. ^e^ 1-year Δ LSM > 25.5% in respect to <25.5%. ^f^ Post-SVR LSM ≥ 20 kPa in respect to LSM < 10 kPa. ^g^ ARFI (acoustic radiation force impulse) elastography. ^h^ Magnetic resonance elastography. ^i^ Child–Pugh B in respect to Child–Pugh A. ^j^ Post-SVR ΔLSM < 30% in respect to ≥30%. ^k^ Annual incidence of HCC is among patients with cirrhosis. ^l^ *Wisteria floribunda* agglutinin positive Mac-2 binding protein.

**Table 2:** Studies evaluating the ability of LSM measured by different types of elastography to predict the risk of HCC occurrence after HCV eradication by DAAs and cut-off values (either baseline or post-therapy) that define an increased or decreased risk of HCC development.

1st Author	Ref.	N	Age (Years)/Males	Type of Elastography	Baseline Cut-Off of LSM	Post-SVR Cut-Off of LSM	HR (95% CI)
Degasperi	42	505	64/60%	TE	>30 kPa	-	NA
Alonso	45	993	61.7/56%	TE	≥17.3 kPa	ΔLSM < 25.5%	NA
Pons	48	572	63.7/49%	TE	-	<10 kPa	0.33 (0.11–0.96)
Tachi	50	233	71.4/46%	ARFI	1.73 m/s	-	8.35 (1.6–43.09)
Tamaki	51	346	68.2/36%	MRE	-	3.75 kPa	3.51 (1.24–9.99)
Rinaldi	52	258	68/55%	TE	27.8 kPa	-	NA
Ravaioli	53	139	62/65%	TE	-	Δ LSM < 30%	5.3 (1.5–18.4)
Shili	55	799	61/52%	TE	>12 kPa	-	14.0 (1.9–109.9)

LSM, liver stiffness measurement; HCC, hepatocellular carcinoma; HCV, hepatitis C virus; DAAs, direct-acting antivirals; Ref., reference; SVR, sustained virological response; HR, hazard ratio; TE, transient elastography; NA, not available; ARFI, acoustic radiation force impulse; MRE, magnetic resonance elastography.

**Table 3:** Different risk categories for HCC in patients with chronic hepatitis C and advanced fibrosis/cirrhosis after viral eradication ^1^. Proposals for surveillance strategies for HCC, according to risk.

Risk for HCC	Patients	Surveillance Strategy
Low	Women younger than 55 years with F3 fibrosis aMAP risk score < 50	Annual US + AFP ^2^ every 6 months
Medium	Cirrhosis Men older than 55 years with F3 fibrosis	US + AFP 2 every 6 months
High	Cirrhosis plus ○ baseline FIB4 > 3.25 ○ post-SVR FIB4 > 3.25 ○ post-SVR AFP > 7 ng/mL ○ low baseline albumin levels ○ low baseline platelet count ○ elevated baseline LSM ^3^	AMRI every 6 months or alternation of AMRI with US every 6 months

^1^ In patients without a second cause of liver disease. ^2^ The best cut-off of AFP should be re-evaluated, given that specificity will improve in patients with viral eradication. To improve sensitivity, the cut-off should be reset to a lower level than 20 ng/mL. ^3^ The best cut-off not well defined. HCC, hepatocellular carcinoma. US, hepatic ultrasound. AFP, alpha-fetoprotein. SVR, sustained virological response. LSM, liver stiffness measurement. AMRI, abbreviated magnetic resonance imaging.

## Abbreviations

The following abbreviations are used in this manuscript:

HCV	hepatitis C virus
HCC	hepatocellular carcinoma
DAAs	direct-acting antivirals
AFP	alpha-fetoprotein
MRI	magnetic resonance imaging
SVR	sustained virological response
USA	United States of America
EASL	European Association for the Study of the Liver
US	ultrasound
cACLD	compensated advanced chronic liver disease
LSM	liver stiffness measurement
ARFI	acoustic radiation force impulse
HR	hazard ratio
ROC	receiver operating characteristic
CHB	chronic hepatitis B
MASLD	metabolic dysfunction-associated fatty liver disease
CAP	controlled attenuation parameter
MASH	metabolic dysfunction-associated steatohepatitis
GLP-1 RA	glucagon-like peptide-1 receptor agonists
FU	follow-up
AMRI	abbreviated magnetic resonance imaging
NC-AMRI	non-contrast AMRI
DEC-AMRI	dynamic extra-cellular contrast-enhanced AMRI
HBP-AMRI	hepatobiliary phase contrast-enhanced AMRI
